# Not all geriatric cachexia is cancer – The difficult lateonset rheumatoid arthritis

**DOI:** 10.1515/rir-2024-0009

**Published:** 2024-03-31

**Authors:** Ana Rubim Correia, Inês Clara, Sara Raquel Martins, Tomás Fonseca

**Affiliations:** Internal Medicine Service, Unidade Local de Saúde de Santo António (ULSSA), Porto, Portugal; Clinical Immunology Unit, Unidade Local de Saúde de Santo António (ULSSA), Porto, Portugal

Dear Editor,

Late-onset rheumatoid arthritis (LORA) refers to the onset of rheumatoid arthritis (RA) in individuals aged 60–65 or older, representing 10%–30% of the total RA population. The increasing of life expectancy over the last two decades will turn LORA a more prominent form of RA. The goal of treating LORA remains consistent with that for younger patients, however, there are several challenges linked with the diagnosis and management of this disease: the need to ruling out other conditions with similar clinical presentations; the absence of specific guidelines for treating RA in this age group; as well as the complexity of selecting appropriate therapies for these patients. The authors believe it is of utmost importance to address issues related to the diagnosis and management of LORA, considering the increasing prevalence of this condition and the absence of specific guidelines for it. We present a case of a patient with LORA to illustrate the difficulties in its management. We believe that more attention should be given to this topic to assist other healthcare professionals who may need to address it and to encourage further research in this area.

**Figure 1:**
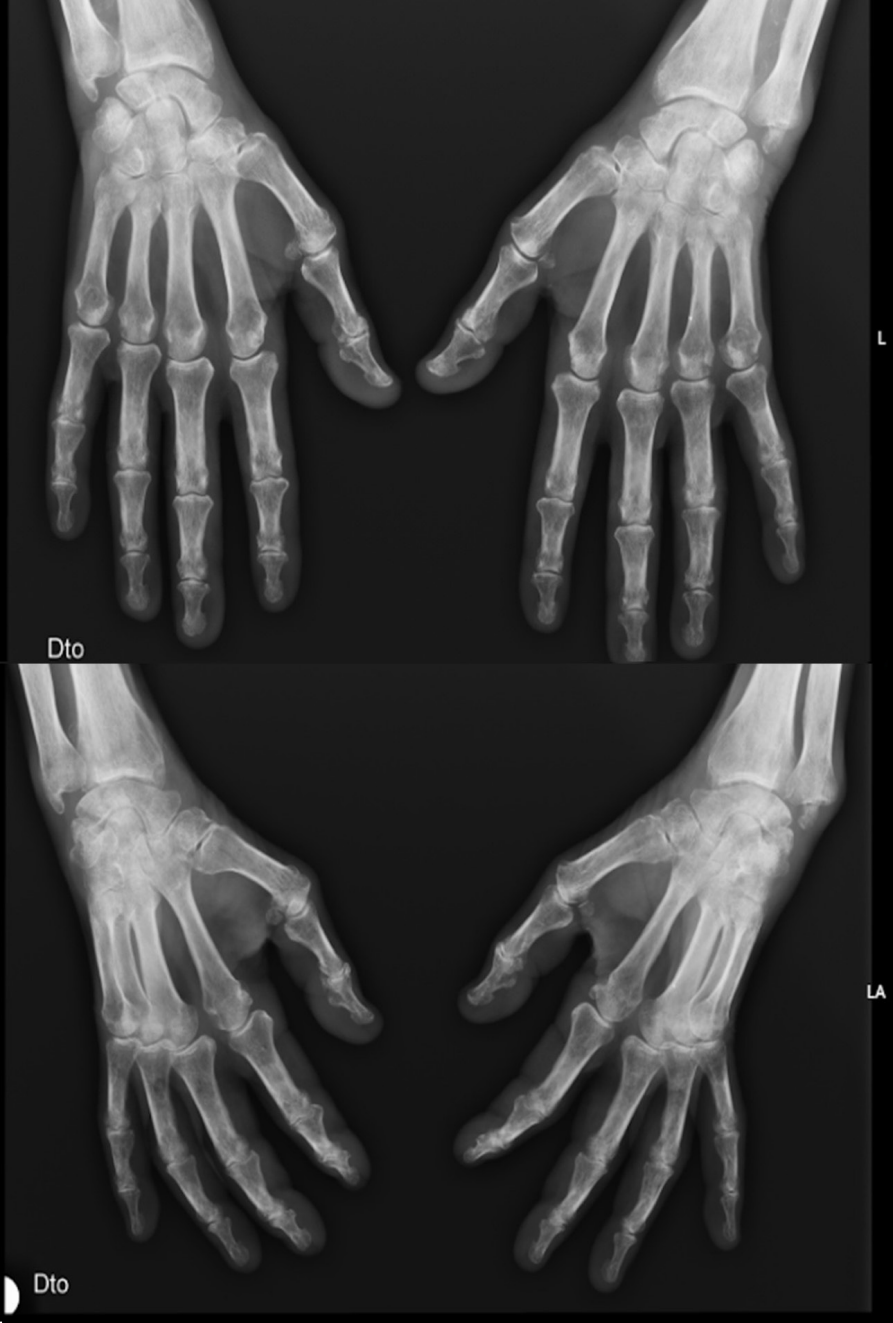
*Radiographs of the hands (posteroanterior, superior; oblique, inferior)*.

A 76-year-old man with hypertension and hypothyroidism went to the Emergency Department (ED) due to a 3-months history of weight loss (15% of the total body weight), asthenia and 1-hour morning stiffness. He reported no fever or night sweats. General examination revealed poor tolerance to any activity, severe muscle mass loss was evident; Body mass index (BMI) was 19.1 kg/m^2^. Several joints were painful, with symmetrical synovitis of the knees, elbows, wrists, proximal interphalangeal and metacarpophalangeal joints (Figure 1 demonstrate the impairment to the hand’s joints). Blood levels revealed inflammatory anemia (Hemoglogin 7.6 g/dL, reference range 12–15) and high inflammatory biomarkers (Erythrocyte sedimentation rate 96 mm/h, reference range 0–25; C-reactive protein 97.7 mg/dL, reference range 0–5; ferritin 757 μg/L, reference range 2.2–178). Blood and urine cultures were negative, and human immunodeficiency, B/C hepatitis viruses and syphilis serologies were negative too. Thyroid function were normal. Transthoracic echocardiogram excluded endocarditis. Full body computed tomography, positron emission tomography, upper and lower digestive endoscopies excluded neoplastic causes. The study was complemented with rheumatoid factor (RF) and anti-citrullinated peptide antibodies (anti-CCP), both elevated (279.9 UI/mL, reference range 0–20, and 435 UI/mL, reference range 0–20, respectively).

**Figure 2:**
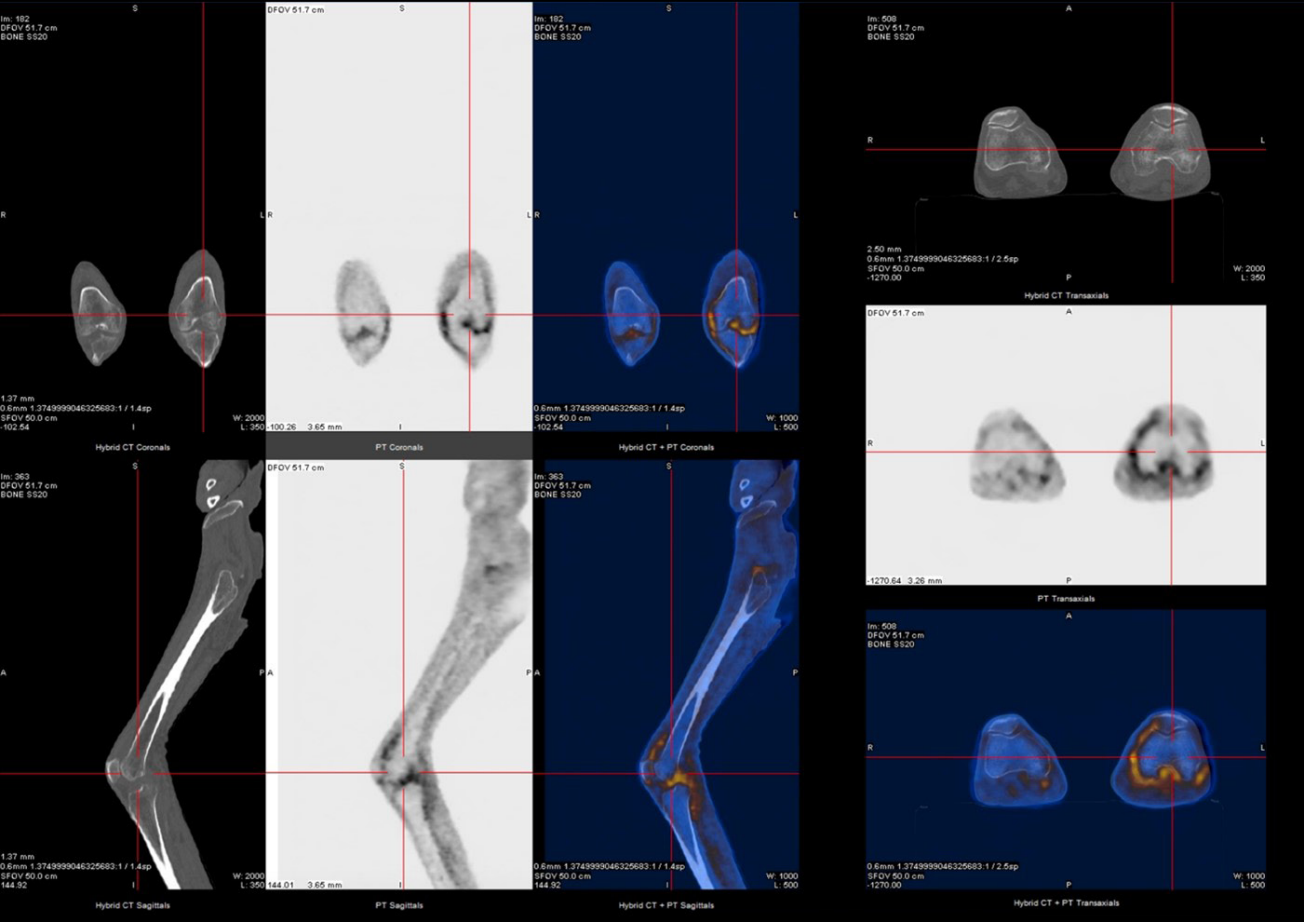
*18F-FDG and PET-CT study reveals slightly increased uptake of FDG in the knee joints (more in the left knee joint, with apparent extension to the subquadricipital synovial bursa region). PET-CT, pistron emission tomography-computed tomography*.

A very active and systemic LORA was assumed, and the patient started on systemic corticosteroids and methotrexate. Initial clinical response was observed with clinical improvement. Nevertheless, a septic left knee arthritis with bacterie-mia led to a long hospital stay (Figure 2). It was resolved after temporary suspension of immunosuppressive therapy, surgery and intravenous (IV) antibiotic therapy. After restarting immunosuppressive therapy, he was admitted to the immunology outpatient clinic for follow-up and started ambulatory motor rehabilitation. However, he soon returned to the ED due to diarrhea, mucositis and pancytopenia secondary to methotrexate toxicity, possibly due to erroneous intake. The condition was resolved after discontinuation of the drug, but a rapid worsening of the arthritis lead to IV infliximab initiation to assure correct drug administration. Six months after infliximab initiation the patient is on clinical remission under outpatient follow-up.

In brief, patients with LORA will differ from patients with early--onset RA in several aspects and should have a personalized therapeutic approach. Clinical presentation is often more acute and severe, with involvement of large joints, higher disease activity and functional impairment. Constitutional symptoms can be the predominant feature, mimicking neoplasms or occult infections, and delaying the correct diagnosis due to the necessary investigational steps to exclude these entities.^[1,2,3,4,5]^ The treatment objectives are transversal in all cases of RA. However, elderly patients often experience more comorbidities and a higher incidence of adverse drug reactions. There are no specific treatment guidelines to LORA and, although the current RA guidelines advocate conventional synthetic disease-modifying antirheumatic drugs (csDMARDs) as first-line therapies, in these group of patients, early biological drugs should be considered. Not only due to the several specific RA poor prognostic factors presented in LORA patients, but also because of the potential of csDMARDs to worsen chronic diseases. This clinical case illustrates some of these subjects. The data regarding the use of biologic or targeted synthetic disease-modifying antirheumatic drugs (b/tsDMARDs) in elderly patients are limited. However, they appear to be as effective and well-tolerated in these patients as in those with early-onset RA. Some literature indicates that b/tsDMARDs do not exhibit higher rates of side effects when compared to csDMARDs. In this case, IV Infliximab (TNF-α inhibitor) turned out to be a logic option, controlling as early as possible the LORA activity.^[6,7,8,9,10]^

Our clinical case highlights how LORA clinical and therapeutic approach can be challenging. A high level of suspicion and early action are essential to delay the progression of the disease and its many complications.
